# In Vitro Antioxidant and Antibacterial Activities of Ethyl Acetate Extracts of *Ziziphus lotus* Leaves and Five Associated Endophytic Fungi

**DOI:** 10.3390/microorganisms12122671

**Published:** 2024-12-23

**Authors:** Amel Ghazi-Yaker, Bart Kraak, Jos Houbraken, El-hafid Nabti, Cristina Cruz, Noria Saadoun, Karim Houali

**Affiliations:** 1 Natural Resources Laboratory, Faculty of Biological and Agronomic Sciences, Mouloud Mammeri Univesity, Tizi-Ouzou 15000, Algeria; amel.ghazi@ummto.dz (A.G.-Y.); noria.saadoun@ummto.dz (N.S.); 2 Laboratory of Analytic Biochemistry and Biotechnology (LABAB), Department of Biochemistry and Microbiology, Faculty of Biological and Agronomic Sciences, Mouloud Mammeri University, Tizi-Ouzou 15000, Algeria; 3 Westerdijk Fungal Biodiversity Institute, Uppsalalaan, 3584 CT Utrecht, The Netherlands; b.kraak@wi.knaw.nl (B.K.); j.houbraken@wi.knaw.nl (J.H.); 4 Laboratoire de Maitrise des Energies Renouvelables, Faculté des Sciences de la Nature et de la Vie, Université de Bejaia, Bejaia 06000, Algeria; el-hafid.nabti@univ-bejaia.dz; 5 cE3c—Center for Ecology, Evolution and Environmental Changes & CHANGE—Global Change and Sustainability Institute, Faculdade de Ciências da Universidade de Lisboa Campo Grande, 1749-016 Lisboa, Portugal; cmhoughton@fc.ul.pt

**Keywords:** *Ziziphus lotus*, endophytic fungi, phenolic compounds, secondary metabolites, antioxidant, antibacterial, GC–MS, *Aspergillus cavernicola*

## Abstract

The exploration of new pharmacological compounds from endophytic fungi offers infinite possibilities. The aim of this study was to evaluate the antibacterial and antioxidant activities of extracts from the leaves of *Ziziphus lotus* and five of its endophytic fungi and investigate the chemical diversity of the secondary metabolites produced. Isolated, purified, and molecularly identified endophytes and plant leaves were subjected to ethyl acetate extraction. The antibacterial potential of the extracts was assessed by the disc diffusion method against five bacterial strains: *Staphylococcus aureus* ATCC 25923; *Staphylococcus aureus* MU50; *Enterococcus faecalis* WDCM00009; *Escherichia coli* ATCC 25922; and *Pseudomonas aeruginosa* ATCC 27853. DPPH and reducing power tests were performed to assess antioxidant potential. GC–MS analysis was used to identify volatile compounds in extracts. Fungal endophytes were identified as *Aspergillus cavernicola*, *Aspergillus persii*, *Alternaria alternata*, *Cladosporium asperlatum,* and *Fusarium incarnatum–equiseti complex*, with respective accession numbers DTO 412-G6, DTO 412-I5, DTO 413-E7, DTO 412-G4, and DTO 414-I2. GC–MS analysis revealed a large number of bioactive compounds. All extracts showed antibacterial activity against at least two of the bacteria tested, and most showed antioxidant activity. The *Aspergillus cavernicola* extract stood out for its higher phenolic content and higher antioxidant and antibacterial activities in all tests.

## 1. Introduction

Recently, research into developing new safe drugs from natural resources has been explored in depth [1]. Medicinal plants have been used to isolate and characterize bioactive metabolites directly. However, the discovery of endophytic fungi within these plants with the ability to produce the same compounds as the host plant or even different metabolites has shifted the focus of new drug sources from plants to fungi [2]. Endophytic fungi colonize the inter- and intra-cellular tissues of plants without causing any apparent damage or disease symptoms [3]. These fungi are found in almost all plants in natural ecosystems and have complex mutual ecological relationships with their host plants [4]. These fungi are capable of producing heterogeneous low molecular weight compounds, also known as secondary metabolites [5]. These are an abundant and valuable source of molecules with a wide variety of chemical structures and biological activities [6], including antitumor, anti-inflammatory, antibacterial, antiviral, antioxidant, and anti-angiogenic [7,8]. Active compounds isolated from endophytic fungi include penicillin, an antibiotic produced by the species *Penicillium notatum* [9], taxol, and vincristine, recognized antineoplastic agents produced by the endophytic fungi *Taxomyces andreanae* and *Fusarium oxysporum* [10].

Currently, serious infections caused by bacteria and their resistance to antibiotics are one of the greatest threats to human health [11]. In addition, oxidative stress is caused by free radicals and is involved in pathophysiological events, including some human diseases such as diabetes mellitus, aging, atherosclerosis, Alzheimer’s disease, and Parkinson’s disease [12]. Exploring natural sources for new bioactive agents may provide leads or solutions for the discovery and development of new drugs [13]. Endophytic fungi play an important role in the search for natural bioactive compounds and could represent an alternative source for the production of therapeutic agents that are difficult to obtain by chemical synthesis. The exploitation of new pharmacological compounds from endophytes is infinite [14].

*Ziziphus lotus* L. (Desf), commonly known as Sedra, is a medicinal plant belonging to the *Rhamnaceae* family, abundant in the Mediterranean region, from Libya to Morocco, Algeria, and Southern European countries such as Spain, Italy, Greece, and Cyprus [15]. The antioxidant, immunosuppressive [15], antibacterial [16], antifungal [17], anti-inflammatory, analgesic [18], and antiulcer [19] properties of this plant have been demonstrated in previous studies.

In this study, we were interested in the isolation and identification of endophytic fungi from the leaves of *Z. lotus*, the evaluation of the antibacterial activity of ethyl acetate extracts of isolated fungi and leaves against human pathogens, and the evaluation of the antioxidant activity of the two types of extracts and finally their analysis by GC–MS.

## 2. Materials and Methods

### 2.1. Plant Material

The leaves of *Z. lotus* were collected from eight healthy, randomly selected plants in the region of Djebla, Tizi-Ouzou, Northern Algeria, in March 2016. Leaves were stored at 4 °C and processed within 48 h of their collection for the isolation of endophytic fungi. Moreover, a quantity of leaves is dried and powdered.

### 2.2. Isolation and Identification of Endophytic Fungi

Endophytic fungi were isolated from the leaves of *Z. lotus*, as described by Ghazi-Yaker et al. (2023) [20]. Five strains belonging to some of the most popular genera of endophytic fungi (*Aspergillus*, *Fusarium*, *Cladosporium*, and *Alternaria*) were selected for this study. Molecular identification was carried out using the ITS region and a reference GenBank’s database as described by Ghazi-Yaker et al. (2023) [20]. The sequences were compared on GenBank using BLAST and in the in-house sequence database of Westerdijk Fungal Biodiversity Institute (Utrecht, The Netherlands).

### 2.3. Secondary Metabolites Extraction

#### 2.3.1. Secondary Metabolites Extraction from *Z. lotus* Leaves

A total of 10 g of air-dried leaf powder was placed in 100 mL of ethyl acetate on a rotary shaker at 350 rpm for 8 h. A filtration through a sterile compress was carried out, followed by a centrifugation at 4000 rpm for 15 min, and the solvent was evaporated using a rotary evaporator at 40 °C. The extracted residue was dissolved in dimethyl sulfoxide (DMSO 10%) to a final concentration of 20 mg/mL, sterilized by filtration through 0.22 µm Millipore filters, and stored at 4 °C to be used as a stock solution for studying the biological activities.

#### 2.3.2. Secondary Metabolites Extraction from Fungal Endophytes

The secondary metabolite extraction from fungi was performed according to the protocol used by Lee et al., 2019 [21]. Endophytic fungi were cultured on potato dextrose agar (PDA) at room temperature for seven days. Five pieces of pure mycelial agar plugs were inoculated aseptically into a 500 mL Erlenmeyer flask containing 100 mL of previously autoclaved potato dextrose broth (PDB). After fermentation (30 days and at room temperature with periodic stirring at 150 rpm for 1 to 2 h), the culture broth was separated from the mycelia by filtration. Culture filtrates were extracted three times with ethyl acetate. The organic phase was collected by decantation and then concentrated under pressure by evaporation of the solvent using a rotary evaporator at 40 °C. The dried extracts were dissolved in dimethyl sulfoxide (DMSO 10%) to a final concentration of 20 mg/mL, sterilized by filtration through 0.22 µm Millipore filters, and stored at 4 °C to be used as a stock solution for studying the biological activities.

### 2.4. In Vitro Biological Activities Testing

#### 2.4.1. Antibacterial Activity

##### Disc Diffusion Assay

The ethyl acetate extracts of plants and fungi were screened for antibacterial activity against five human pathogenic bacteria. They contained both Gram-positive (*Staphylococcus aureus* ATCC 25923, *Staphylococcus aureus* MU50, and *Enterococcus faecalis* WDCM00009) and Gram-negative bacterial strains (*Escherichia coli* ATCC 25922 and *Pseudomonas aeruginosa* ATCC 27853). All of the bacteria in this experiment were supplied by the microbiology laboratory of MOULOUD MAMMERI University (Tizi-Ouzou, Algeria).

Before testing, the bacteria strains were sub-cultured in Petri dishes containing nutrient agar. After 18 h of incubation at 37 °C, suspensions with an optical density between 0.08 and 0.1 at λ = 625 nm were prepared for each microorganism in sterile physiological water. The agar disc diffusion method [22] was used to investigate the antibacterial activity of all ethyl acetate extracts. For this purpose, the freshly prepared bacterial suspensions are swabbed on sterile Muller–Hinton Petri dishes. Sterilized Whatman paper discs (6 mm) containing 25 μL of each ethyl acetate extract were placed on the surface of the seeded Muller–Hinton. The plates were kept at 4 °C for two hours to facilitate the diffusion of the secondary metabolites from discs, and then they were incubated at 37 °C. After 24 h of incubation, the presence around the discs of a circular inhibition zone in which there is no growth of microorganisms denotes their sensitivity to this extract. The diameter of the inhibition zones is measured using a ruler. The larger the zone of inhibition, the more sensitive the bacteria. Chloramphenicol discs (30 μg) are used as a positive control. Negative controls were prepared with the same solvent used to dissolve the extracts (DMSO 10%). All experiments were carried out in triplicate (n = 3), and results are reported as mean ± SD values.

##### Determination of Minimum Inhibitory Concentration (MIC) and Minimum Bactericidal Concentration (MBC)

The micro-dilution method [23] is used to determine the minimum inhibitory concentration (MIC). The MIC values were determined for the bacterial strains that were sensitive to the extracts in the disc diffusion assay. The inocula of the bacterial strains were prepared from 10-hour-old broth cultures, and suspensions were adjusted to 0.5 McFarland standard turbidity. Dilution series ranging from 10 to 0.004 mg/mL in Muller–Hinton Broth (MHB) are prepared. In each well of a 96-well plate, 20 μL of the bacterial strain is applied, 60 μL of Muller–Hinton Broth, and 80 μL of a dilution of extract already prepared. Thus, the dilutions are reduced by half and go from 5 to 0.002 mg/mL. Wells serving as negative control do not contain extract, and those serving as positive control contain the extract without bacterium. The plates are then incubated at 37 °C for 18 h. For the detection of the MIC, 5 μL of resazurin (0.015%) is added to each well. The MIC is the smallest concentration of the extract, which does not produce a change in resazurin color and which corresponds to the absence of bacterial growth.

MBC (minimum bactericidal concentration) is the minimum concentration of the extract capable of killing the inoculum. It is determined by inoculating on Muller–Hinton Agar medium, 10 µL aliquots taken from the wells where there has been no change in the color of the resazurin and the MBC corresponds to the smallest concentration, which gives no subculture.

#### 2.4.2. Antioxidant Assays

Two different assays, including free radical scavenging DPPH assay and reducing power assay, were used to evaluate the antioxidant potential of the endophytic fungal extracts. The total phenolic content was tested by Folin–Ciocalteau reagent-based assay.

##### DPPH (1,1-Diphenyl-2-Picrylhydrazyl) Assay

The evaluation of the scavenging power of the extracts is carried out according to the protocol described by Yadav et al., 2014 [24]. Concentrations of fungal and plant extract (10, 30, 50, 100, 200, 300, 400, 600 µg/mL) in DMSO 10% and DPPH (0.1 mM) in ethanol were prepared. Different concentrations of ascorbic acid were used as the standard. A total of 2 mL of DPPH solution was added to the different test samples (0.2 mL) and incubated in darkness for 30 min at room temperature. The absorbance was measured at 517 nm by a Spectrophotometer UVmini-1240 (Shimadzu, Suzhou, Jiangsu, China). The ethanol solvent is used as a blank, and the absorbance of the DPPH radical without antioxidants served as the control. The percentage inhibition was calculated according to the following equation:

% inhibition = 100 × [(Absorbance of control – Absorbance of sample)/Absorbance of control]

The amount of sample necessary to decrease the absorbance by 50% of DPPH (IC50) was calculated graphically by linear regression. All experiments were carried out in triplicate, and results are reported as mean values.

##### Reducing Power Assay

The evaluation of the reductive potential of the extracts is carried out according to the protocol described by Oyaizu (1986) [25]. A total of 1 mL of the concentration (1 mg mL^−1^) of samples and standard was mixed with phosphate buffer (2.5 mL, pH 6.6), and potassium ferricyanide (K_3_Fe (CN)_6_) (2.5 mL, 1%) was added to the mixtures. After incubation at 50 °C for 20 min in a water bath, the reaction was stopped by the addition of 2.5 mL of 10% trichloroacetic acid. The mixture was then centrifuged at 1000× *g* for 8 min. A total of 2.5 mL of the supernatant was mixed with the same volume of distilled water and 0.5 mL of a 0.1% iron chloride (FeCl_3_) solution. The absorbance was measured at λ = 700 nm against a similarly prepared blank, replacing the test compound with DMSO 10%, which allowed for the calibration of the spectrophotometer UVmini-1240 (Shimadzu, Suzhou Jiangsu, China). A high absorbance value indicates greater reducing power. Ascorbic acid was used as standard. All experiments were performed in triplicates. Results were reported as mean ± SD values.

##### Determination of Total Phenolic Content

The total phenol content of ethyl acetate extracts was estimated using the Folin–Ciocalteu reagent-based assay using gallic acid as standard [24]. To 1 mL of each extract (1 mg/mL), 500 µL of (50%) Folin–Ciocalteu reagent was added, followed by the addition of 1.5 mL of 20% Na_2_CO_3_. The final volume was 5 mL made by adding distilled water. The mixture was incubated at room temperature for 30 min, and the absorbance of the developed color was recorded at 765 nm using a spectrophotometer UVmini-1240 (Shimadzu, Suzhou Jiangsu, China). The same procedure was repeated with 1 mL aliquots of 10 to 70 µg/mL gallic acid solutions in DMSO 10% used as standard for the calibration curve. The total phenolic value was obtained from the regression equation and expressed as mg/g gallic acid equivalent.

### 2.5. Identification of Bioactive Constituents by GC–MS

Analysis of volatile compounds was performed using GC–MS. It is carried out on a chromatograph MASTER GC- Fast Gas Chromatograph System (DANI Instruments, Milan, Italy) coupled to a MASTER TOF MS A-Plus mass spectrometer (DANI Instruments, Milan, Italy) with electronic impact with an ionization energy of 70 eV. The chromatograph is equipped with an HP-1MS capillary column (30 m × 0.25 mm × 0.25 µm). The stationary phase consists of 1% Phenyl and 99% dimethylpolysiloxane. The carrier gas is pure helium 6.0 with a flow rate of 0.5 mL/min. The column is programmed at the following temperatures: 70 °C for 5 min; then, an increase of 10 °C/min to 300 °C is carried out. The volume of the injected solution is 1 µL in Splitless mode. The temperature of the interface and the source is 200 °C.

Chromatograms and mass spectra were recorded. Compounds are detected according to their order of elution. They are identified using their retention time and their mass spectrum compared to data from the NIST (National Institute of Standards and Technology, Gaithersburg, MD, USA) library. The identified compounds were then subject to a bibliographic search for their biological activities.

### 2.6. Statistical Analysis

All values are expressed as arithmetic means ± standard deviations (SD). The statistical significance of any difference in each parameter among the antioxidant essays was evaluated by analysis of variance (ANOVA) using Stat Box 6.40 software. *p*-values of less than 0.05 were considered statistically significant.

## 3. Results

### 3.1. Fungal Strains Identification

Five species were identified as follows:

*Cladosporium asperulatum*: Identification based on partial actin and elongation factor 1-alpha sequences. DTO 412-G4 has a 100% similarity with actin and TEF (Act, 225/225 nt; TEF, 431/431 nt) sequences generated from the type of *Cladosporium asperulatum*;

*Aspergillus cavernicola*: Identification based on partial tubulin sequence. DTO 412-G6 has a 100% (422/422) similarity with the BenA sequence generated from the *A. cavernicola* type strain (CBS 117.76, EF652332);

*Aspergillus persii*: Identification based on partial tubulin sequences. DTO 412-GI5 has a 99.6% (546/548) similarity with the BenA sequence generated from the *A. persii* type strain (MUCL 41970, AY819988);

*Alternaria alternata*: More barcodes are needed to confidentially identify this strain at the species level. Identification based on partial ITS and TEF sequences;

*Fusarium incarnatum equiseti complex*: Identification based on ITS. In order to obtain a better ID, a TEF and RPB2 sequence needs to be generated.

### 3.2. Antibacterial Potential of Extracts

**Figure 1:**
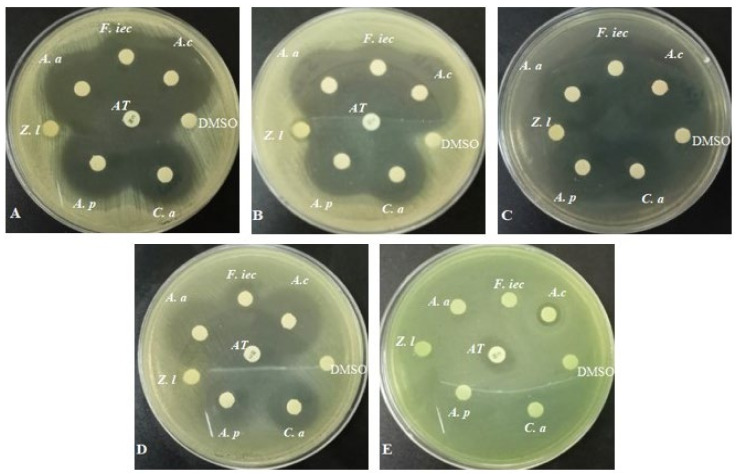
Antibacterial activity of ethyl acetate extracts (**A**) *S. aureus* ATCC 25923, (**B**) *S. aureus* MU50, (**C**) *E. faecalis* WDCM00009 (**D**) *E. coli* ATCC 25922, and (**E**) *P. aerugenosa* ATCC 27853 (F.iec: *Fusarium incarnatum–equiseti* complex; *A.c: Aspergillus cavernicola*; *A.p: Aspergillus persii*; C.a: *Cladosporium asperlatum*; A.a: *Alternaria alternata*; Z.l: *Ziziphus lotus*; DMSO: Dimethylsulfoxide; AT: antibiotic Chloramphenicol).

The antibacterial activity of endophytic fungi and leaf extracts was tested in vitro by disc diffusion (Figure 1, Table 1). The leaf extract of *Z. lotus* showed no inhibitory activity against the bacteria tested, with the exception of strains of *S. aureus*, with which a weak zone of inhibition was noted (8 mm).

All the extracts of endophytic fungi isolated from *Z. lotus* had inhibitory effects on at least four of the bacteria tested. The zones of inhibition obtained varied from 9 to 32 mm. The fungal extracts were more effective on Gram-positive bacteria than on Gram-negative bacteria. In fact, of the two Gram-negative strains used, the fungal extracts were more active against *E. coli* ATCC 25922 with a larger inhibition zone of 25 mm obtained with *A. cavernicola*, whereas for *P. aeruginosa* ATCC 27853, only the *A. cavernicola* extract showed activity.

The *A. cavernicola* extract was the only one capable of inhibiting the five bacteria tested. It is also remarkable that the largest zones of inhibition were obtained with this same extract against the Gram-positive bacteria tested (32, 31, 33, and 30 mm against *S. aureus* ATCC 25923, *S. aureus* MU50, and *E. faecalis* WDCM00009, respectively). These zones were also larger than those obtained with the standard antibiotic used (31 mm, 25 mm, and 25 mm, respectively).

The resulting MIC and MBC of the five endophytic fungi crude extracts against tested pathogenic bacteria are depicted in Table 2.

Interestingly, *A. cavernicola* showed a broad spectrum of antibacterial activity, with the MIC ranging from 0.078 to 0.625 mg/mL. The MIC test indicated that the concentration of *A. cavernicola* extract required to prevent the growth of the test microbes was found to be as follows: 0.078 mg/mL against *E. coli* ATCC 25922, *E. faecalis* WDCM00009, and *S. aureus* ATCC 25923; 0.312 mg/mL against *S. aureus* MU50; and 0.625 mg/mL against *P. aeruginosa* ATCC 27853. The MBC of extract against all the microbes ranged from 1.25 to 5 mg/mL. Aligiannis et al. (2001) [26] proposed a classification of MICs, which stated that strong inhibitors had MIC values < 0.5 mg/mL, moderate inhibitors had MIC values between 0.6 and 1.5 mg/mL, and weak inhibitors had MIC values above 1.6 mg/mL. Based on this classification, *A. cavernicola* reveals a moderate activity against *P. aeruginosa* ATCC 27853 and a strong activity against the remaining four strains. *A. persii* exhibited strong activity against *E. faecalis* WDCM00009 (0.078 mg/mL), *S. aureus* ATCC 25923, and *E. coli* ATCC 25922 (0.312 mg/mL), and moderate activity against *S. aureus* MU50.

On the other hand, *A. alternata* exhibited strong activity (0.312 mg/mL) against both *S. aureus* used and moderate activity against *E. faecalis* WDCM00009 and *E. coli* ATCC 25922 (0.625 mg/mL). *F. incarnatum–equiseti complex* exhibited moderate activity against the tested bacteria in the range of 0.625–1.25 mg/mL. The MBC values of *Fusarium* were greater than 5 mg/mL. *C. asperlatum* showed strong activity against *E. coli* ATCC 25922 (0.312 mg/mL) and moderate activity against *S. aureus* ATCC 25922 (1.25 mg/mL). Remarkably, all extracts of endophytic tested inhibited the growth of *S. aureus* MU50 (hospital-acquired MRSA, vancomycin-intermediate *S. aureus*, VISA) with MIC of 0.312 mg/mL for *A. cavernicola* and *A. alternata*, 1.25 mg/mL for *F. incarnatum–equiseti complex* and *A. persii* and 2.5 mg/mL for *C. asperlatum,* respectively.

### 3.3. Antioxidant Potential of Extracts and Phenolic Content

#### 3.3.1. DPPH Assay

**Figure 2:**
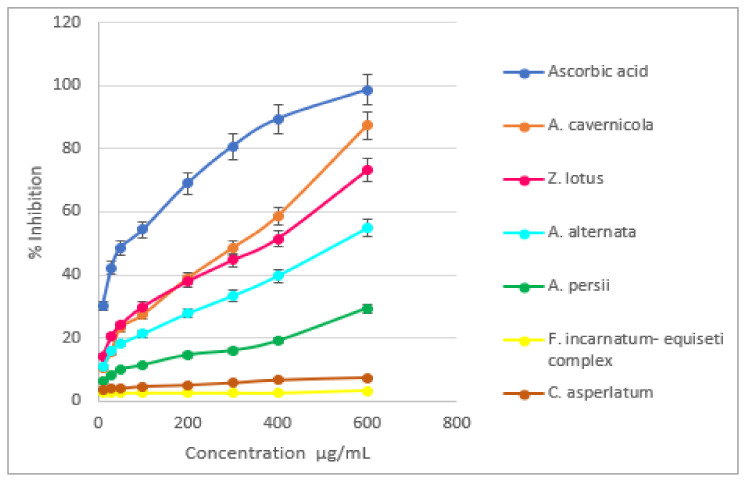
DPPH radical scavenging activities of endophytic fungi and *Z. lotus* leaves ethyl acetate extracts in comparison with ascorbic acid.

The reaction was visible as a color change from purple to yellow. Concerning the ascorbic acid, the leaf extract, and the fungal extract of *A. cavernicola*, *A. alternata,* and *A. persii*, increasing concentration showed a dose-response scavenging effect. Concerning *F. incarnatum–equiseti complex* and *C. asperlatum*, none demonstrated DPPH scavenging antioxidant potential (Figure 2). The analysis of variance (ANOVA) between all the extracts showed a very highly significant difference (*p*-value = 0). After ascorbic acid, the extract of *A. cavernicola* exhibited the greatest activity at the lowest IC50 concentration (309.57 ± 7.59 µg/mL), followed by *Z. lotus* (IC50 = 355.68 ± 4.31 µg/mL), *A. alternata* (IC50 = 535.67 ± 3.21 µg/mL), and *A. persii* (IC50 = 1253.59 ± 11.09 µg/mL).

#### 3.3.2. Reducing Power Assay

**Figure 3:**
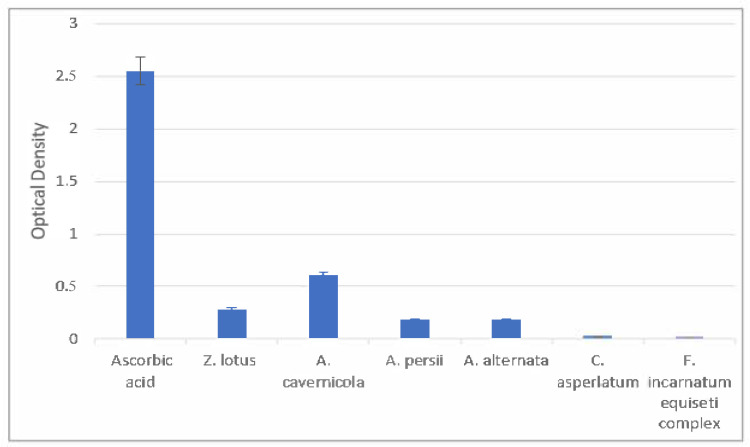
Reducing power activity of samples and ascorbic acid at 1 mg/mL.

The resulting reducing power of endophytic fungi and *Z. lotus* leaf ethyl acetate extracts is shown in Figure 3.

In reducing power assay, reducing ability is measured by a change in Fe ^3+^ to Fe ^2+^. The analysis of variance (ANOVA) between all the extracts showed a very highly significant difference (*p*-value = 0). After ascorbic acid, the extract of *A. cavernicola* presented the highest absorbance values (0.61), followed by *Z. lotus* (0.28), indicating their greater reductive potential and electron donor ability for stabilizing free radicals in comparison with the other extracts, *A. persii* and *A. alternata*, with the same absorbance value of 0.18. Concerning *C. asperlatum* and *F. incarnatum–equiseti complex*, the absorbance values are low (0.02 and 0.01), attesting to the absence of reducing activity.

#### 3.3.3. Determination of Total Phenolic Content

**Figure 4:**
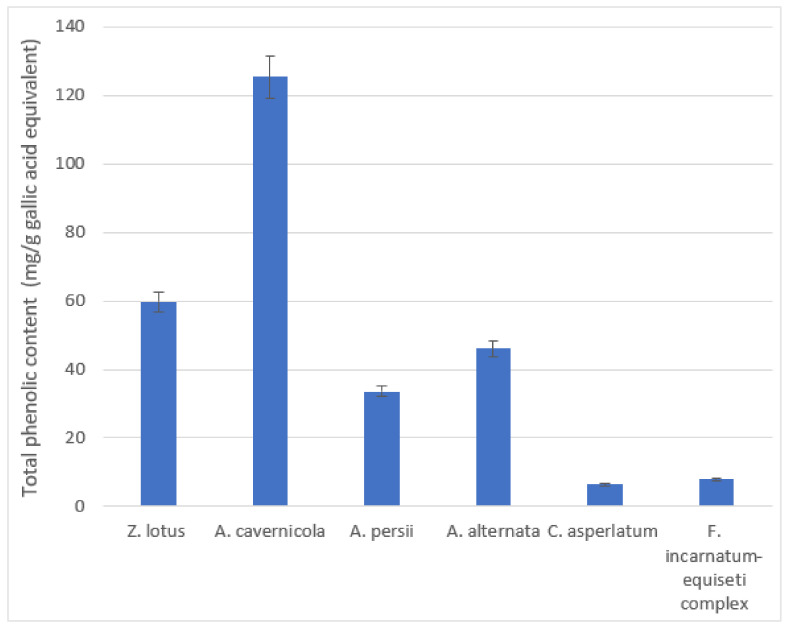
Total phenolic contents in *Z. lotus* and its endophytic fungi.

According to the analysis of variance (ANOVA) obtained for total phenolic compounds, a very highly significant difference (*p*-value = 0) was observed between all the extracts. Concentrations obtained varied from 6.4 to 125.32 mg/g gallic acid equivalent. A higher amount of phenolic compounds was determined and quantified in *A. cavernicola* extract. In fact, *A. cavernicola* contained a twofold higher polyphenol content (125.32 mg/g gallic acid equivalent) in comparison with *Z. lotus* (59.64 mg/g gallic acid equivalent), followed by *A. alternata* (46.07 mg/g gallic acid equivalent) and *A. persii* (33.62 mg/g gallic acid equivalent), whereas *F. incarnatum–equiseti complex* and *C. asperlatum* extracts contained considerably less concentration of phenols (7.9 and 6.4 mg/g respectively) (Figure 4).

### 3.4. Identification of Bioactive Compounds by GC–MS

The GC–MS analysis was used to identify volatile compounds in the extracts. The GC–MS is a unanimously accepted method for the analysis of phyto-constituents. It is also often used in the analysis of fungal extracts. Based on the results of antibacterial and antioxidant activities, the extracts of endophytes *A. cavernicola*, *A. persii*, and *A. alternata* were selected for this analysis in addition to plant extract. GC–MS analysis of all extracts revealed a totality of 72 different compounds, shown in Table 3.

GC–MS analysis conducted on the crude ethyl acetate extract of *A. cavernicola* revealed 26 bioactive compounds and showed the following main compounds: Butylphosphonic acid, 2-ethylhexyl propyl ester (37.468%); 4-tert-Octylphenol, TMS derivative (10.892%); 2,3-Dimethyl-2-heptene (8.4199%); and 5,6-Dihydropenicillic acid (7.1999%). The following compounds are found only in the *A. cavernicola* extract: 2,3-Dimethyl-2-heptene; Hexadecanoic acid, 1-[[[(2-aminoethoxy) hydroxyphosphinyl] oxy] methyl]-1,2-ethanediyl ester; 5-Hydroxy-4-methoxy-5-(prop-1-en-2-yl)furan-2(5H)-one; 2-Nonen-4-one; 2-(4-Ethoxyanilino)-N-propylpropanamide, Ac derivative; Butylphosphonic acid, 2-ethylhexyl propyl ester; 4-tert-Octylphenol, TMS derivative; 1H-Imidazole, 1-(1-oxooctadecyl)-; 2-(1-Methylpiperidin-2-yl) ethanol; 4-(4-Hydroxyphenyl)-4-methyl-2-pentanone, TMS derivative; Sydowinin A, 2TMS derivative; Hexanedioic acid, dioctyl ester; Phenanthrene-10-ethanamine, 3-bromo-á-hydroxy-N,N-diheptyl-; O,O-diphenylphosphate; Cyclodecasiloxane, eicosamethyl.

GC–MS study carried out on the crude ethyl acetate extract of *A. persii* revealed 28 bioactive compounds, the most important being 2,5-furandicarboxylic acid, tetrahydro-, dimethyl ester (21.4686%); phosphinic acid, (1,1-dimethylethyl)[4-(1,1-dimethylethyl)phenyl]- (15.0705%); 2,5-hexadienoic acid, 3-methoxy-5-methyl-4-oxo-(penicillic acid) (11.5193%); l-alanine, N-(3-fluorobenzoyl)-, heptyl ester (10.3966%). The following compounds are found only in the *A. persii* extract: Cyclopentane, 1-acetyl-1,2-epoxy-; Flucytosine; 2-Benzyl-3-methoxycyclopropane carboxylic acid, methyl ester; Succinic acid, ethyl 4-methylhept-3-yl ester; 5-Hydroxy-4-methoxy-3-(1-methoxypropan-2-yl)furan-2(5H)-one; Erythritol, 2,5-Furandicarboxylic acid, tetrahydro-, dimethyl ester; Epinephrine; (á)-, 3TMS derivative; l-Alanine, N-(3-fluorobenzoyl)-, heptyl ester; Phthalic acid, butyl hex-3-yl ester; Ethene-1,1-diamine, 2,2-dinitro-; 3-Cholestanol piperidinomethyl ether; Pentanedioic acid, 1-(6-bromo-9-phenanthrenyl)-2-(diheptylamino)ethyl monoester; Hexasiloxane, tetradecamethyl-.

Moreover, the compounds Penicillic acid (2,5-Hexadienoic acid, 3-methoxy-5-methyl-4-oxo; Succinic acid, ethyl pent-4-en-2-yl ester; 5,6-Dihydropenicillic acid; Cholestan-5-en-3-ol piperidinomethyl ether; and Phosphinic acid, (1,1-dimethylethyl)[4-(1,1-dimethylethyl)phenyl]-) are found only in fungal extracts belonging to the genus *Aspergillus* (*A. cavernicola* and *A. persii*).

GC–MS analysis conducted on the crude ethyl acetate extract of *A. alternata* revealed 31 bioactive compounds. Tenuazonic acid (21.6718%); Tetracosamethyl–cyclododecasiloxane (11.5408%); Bis (2˗ethylhexyl) phthalate (10.6541%); Cyclooctasiloxane, hexadecamethyl- (9.5733%); Cyclononasiloxane, octadecamethyl (8.2317%) are among the most important components. The following compounds are detected only in the *A. alternata* extract: Tenuazonic acid; Tetratetracontane; 8-Cinnamoyl-5,7-dihydroxy-2,2,6-trimethylchromene, 2TMS derivative; Octyl tetracosyl ether; tert-Hexadecanethiol; Hexadecane, 1-bromo-; Heptacosane; Terephthalic acid, bis (2,2,3,3,4,4,5,5,6,6,7,7-dodecafluoroheptyl) ester; 4-Hydroxybenzyl alcohol, 2TBDMS derivative; 2-(5-Bromo-pyridin-2-ylamino)-3,3,3-trifluoro-2-(4-methoxy-benzoylamino)-propionic acid methyl ester; N-(2-Hydroxy-4-nitrophenyl)-4-methoxybenzamide, TMS derivative; Octadecane, 1-iodo-; Hexadecane; Bis(2-ethylhexyl) phthalate; [1,3]-Oxazino[5,6-c]quinoline,3-(3,4-methylenedioxybenzyl)-5-trifluoromethyl-3,4(2H)-dihydro-7-methoxy-; para-Isopropylbenzoic acid trimethylsilylester; Dodecane, 1-iodo-; 1,4-Benzenedicarboxylic acid, bis(2-ethylhexyl) ester; Dodecane, 3-methyl-; Supraene; Phenol, 2-amino-4,6-bis (1,1-dimethylethyl)-; Benzene, 1,1,1-[1-(bromomethyl)-2-methoxy-1-methyl-1-ethanyl-2-ylidene]tris.

The extract of *Z. lotus* leaves contained 17 compounds, of which the maximum quantum was 1,1,1,5,7,7,7-Heptamethyl-3,3-bis (trimethylsiloxy) tetrasiloxane (17.5764%); followed by Cyclooctasiloxane, hexadecamethyl- (16.4283%). Among them, six compounds are also detected in the three fungal extracts (Cyclohexasiloxane, dodecamethyl-; Octasiloxane, 1,1,3,3,5,5,7,7,9,9,11,11,13,13,15,15-hexadecamethyl-; Tetracosamethyl-cyclododecasiloxane; Heptasiloxane, hexadecamethyl-; 1,1,1,5,7,7,7-Heptamethyl-3,3-bis(trimethylsiloxy)tetrasiloxane; and Cyclononasiloxane, octadecamethyl-). The compound Cyclooctasiloxane, hexadecamethyl-, is also detected in *A. persii* and *A. altrnata* extracts. 3,4-Dihydroxymandelic acid, 4TMS derivative, is also detected in *A. cavernicola* and *A. persii* extracts. The 3,4-Dihydroxyphenylglycol, 4TMS derivative; Heptasiloxane, 1,1,3,3,5,5,7,7,9,9,11,11,13,13-tetradecamethyl- are also detected in *A. alternata* extract. Hexanedioic acid, bis(2-ethylhexyl) ester is also detected in *A. persii* extract.

## 4. Discussion

Endophytic microorganisms living inside their host can often be cultured and grown after isolation from their host plants. In culture, outside of their host tissue, endophytic fungi are also known to produce a number of important secondary metabolites. Endophytic fungi are believed to imitate, duplicate, and modify the secondary metabolite compounds of their hosts, which is a complex process [27]. This represents a substantial biotechnological potential of endophytic fungi for the discovery of new drugs [28,29]. Thus, endophytic fungi can be used to isolate active metabolites and reduce the large-scale utilization of plants as a method to protect the environment [30]. Endophytic fungi are advantageous because they have short generation time, high biomass production due to high growth rates, and good handling features in bioreactors [31].

The in vitro antibacterial and antioxidant activities screening of ethyl acetate crude extracts of *Z. lotus* leaves and five endophytic fungi associated with the leaves of this same plant was performed in this study. Ethyl acetate extraction is the most efficient approach to isolating fungal secondary metabolites [32]. As an extraction solvent, ethyl acetate selectively extracts low-molecular-weight phenolic compounds and high-molecular-weight polyphenols [24,33]. Conde et al. (2008) [34] have reported that ethyl acetate allowed for the highest phenolic content and the selective removal of non-phenolic compounds.

Most studies have shown that endophytes were a good source of antibacterial agents [35]. Schulz et al. (2015) [36] suggested that to grow asymptomatically within their plant hosts, fungal endophytes would need to not only maintain a balanced antagonism with their plant host but also with other bacterial and fungal communities in the host. This would explain the synthesis of antibacterial and antifungal metabolites by fungal endophytes. Secondary metabolites are hypothesized to work as antibacterial substances in host plants and endophytic fungi by preventing cell wall development, modifying membrane permeability, inhibiting nucleic acid production, and decreasing the formation of essential metabolites in bacterial cells [37]. Among these substances, the phenolic compounds exhibit great structural diversity, such as the presence of hydroxyl groups, as well as their substitution position and saturated side-chain length, which confers antibacterial activity to these compounds [38,39].

In our study, the foliar extract of *Z. lotus* showed no inhibitory activity against Gram-positive and Gram-negative bacteria used in the tested concentration except for the two *S. aureus*. Yahia et al., 2020, [40] reported that the methanolic extract of *Z. lotus* leaves at a concentration of 10 mg/mL inhibited the growth of *S. aureus*, *Listeria monocytogenes*, *Salmonella typhimurium*, and *E. coli*, with inhibition zones ranging from 10 to 13 mm. Concerning the fungal extracts, we observed that all endophytic fungi extracts showed antibacterial activities against the strains *S. aureus* ATCC 25923, *S. aureus* MU50, *E. faecalis* WDCM00009, and *E. coli* ATCC 25922, whereas only *A. cavernicola* was able to inhibit the growth *P. aeruginosa* ATCC 27853. The fungal extracts were more effective on Gram-positive bacteria compared to Gram-negative bacteria. This may be due to the structural differences presented in the cellular walls of the two types of bacteria. Gram-negative bacteria have an outer polysaccharide membrane, which carries the structural lipopolysaccharide components. This makes the cell wall impermeable to lipophilic solutes. Gram-positive should be more susceptible, having only an outer peptidoglycan layer, which is not an effective permeability barrier [41,42].

Several studies have reported the antibacterial activity of extracts from fungal endophytes. *Cladosporium* spp. extracts reported by other works present antibacterial potential. The ethyl acetate extract of the endophytic *Cladosporium ramotenellum* isolated from *Securinega suffruticosa* exerted significant inhibitory effects on *E. faecalis*, *E. coli*, *P. aeruginosa*, and *S. aureus* [43].

The genus *Fusarium* was reported to produce diverse bioactive secondary metabolites with a broad spectrum of biological activities such as antibacterial [44], antifungal [45], and cytotoxic [46]. Tayung and Jha (2010) [47] reported that crude extracts obtained with ethyl acetate from a fermented broth of *Fusarium* sp. isolated from *Taxus baccata* showed significant antibacterial activity against *S. aureus* and *E. coli*, among others. Generally, *F. incarnatum–equiseti complex* strains are considered moderately virulent to many agricultural crops and produce a variety of mycotoxins, which represent a serious threat to food safety and public health [48]. Our study shows that *F. incarnatum–equiseti complex* can also function as an antibacterial agent. This result is of great significance for the development of new bio-control agents. Moreover, our results are in agreement with the study by Chatterjee et al. (2019) [37], which found that the ethyl acetate extract of endophytic fungus *A. alternata* VN3 isolated from *Vitex negundo* L. was effective against *S. aureus* and *E. coli* and not against *P. aeruginosa.* Abd Elghafar et al. (2022) [49] reported that the ethyl acetate crude extract of *A. alternata* from leaves of *Ziziphus spina-christi* exhibited promising antibacterial activity against Gram-negative bacteria (*P. aeruginosa* ATCC 27853, *E. coli* ATCC 11229, *Proteus vulgaris* RCMB 004, and *Klebsiella pneumonia* RCMB 003) and Gram-positive bacteria (*S. aureus* ATCC 25923, *Bacillus subtilis* RCMB 015, and *Staphylococcus epidermidis* ATCC 14990). Moreover, Kim et al. (2017) [50] reported that the fungus *A. persii* strain NIBRFGC000004109 showed strong antibacterial activity, especially against plant-pathogenic bacteria, including *Agrobacterium tumefaciens*, *Ralstonia solanacearum*, *Xanthomonas arboricola pv. pruni*, and *Xanthomonas oryzae pv. oryzae*. Furthermore, our study reports, for the first time, the antibacterial effect of extract from the endophytic *A. cavernicola.* Our results revealed that the ethyl acetate extract of *A. cavernicola* exhibited broad-spectrum activity in two different groups of bacteria. It was the only one able to inhibit the five tested bacteria.

In our study, extracts with antioxidant potential also had a good amount of total phenols as tested by Folin–Ciocalteu reagent-based assay. Previous studies conclude that there is a linear correlation between total phenolic content and the antioxidant potential of any sample [51]. Nuraini et al. (2019) [52] suggested that the phenolic contents in endophytic fungal culture extracts were the major antioxidant constituents. The low antioxidant capacity in the *C. asperlatum* and *F. incarnatum–equiseti complex* extracts observed in our results may be due to the low content of phenol. Phenolic compounds have hydroxyl groups and have been described for their antioxidant potential due to their ability to eliminate free radicals, high reducing power, and ability to inhibit lipid oxidation [39,53]. The ability of phenolic compounds to act as reducing agents, electron donors, oxygen quenchers, or metal chelators is mainly attributable to their redox characteristics [54]. Compared to the potency of the standard antioxidant ascorbic acid, the foliar and fungal extracts showed lower antioxidant activity, which could be justified by the fact that the antioxidant used as standard is an isolated molecule while the fungal and foliar extracts represent a set of substances at varied concentrations.

The results of this study link with some previous findings on endophytic fungi and their antioxidant activities.

Endophytic fungi have been reported as a good source of antioxidant metabolites [55]. Abd Elghafar et al., 2022, [49] isolated *A. alternata* from leaves of *Z. spina-christi*. Ethyl acetate crude extract of this *A. alternata* was assessed as an antioxidant using the DPPH method. Results revealed that the extract of *A. alternata* exhibited promising antioxidant activity compared to ascorbic acid, where activity at 500–2000 µg/mL was above 50%. In addition, results illustrated that IC50 of ethyl acetate crude extract of *A. alternata* was 409 µg/mL. In our study, the value is 535.67 µg/mL. The crude extract of *A. alternata* isolated from *Coffea arabica* L. showed an IC50 value of 86.7 μg/mL when tested by DPPH free radical scavenging assay [56]. Yadav et al., 2014, [24] isolated 21 endophytic fungi from *Eugenia jambolana* Lam, of which eight were *Aspergillus* (*Aspergillus* sp., *Aspergillus peyronelii*, *Aspergillus niger*, *Aspergillus flavus*, *Aspergillus tubingensis*, *Aspergillus japonicus*, *Aspergillus terreus*, *Aspergillus niger* strain, and *Aspergillus aff. fumigatus*). Ethyl acetate crude fungal extracts were explored for antioxidant potential by using three different methods. They all showed antioxidant activities up to varying extents. *Aspergillus* sp. showed a high antioxidant capacity, followed by *A. peyronelii* and *A. niger*.

The present study also shows that *Z. lotus* exerted an antioxidant activity. Our results agree well with the findings of Dhibi et al., 2022, [57], who have also observed that *Z. lotus* extracts exerted antioxidant effects.

Secondary metabolites from fungi are a plentiful and valuable source of molecules that exhibit a wide range of chemical structures and biological activities [6]. The GC–MS analysis of the ethyl acetate extracts revealed the presence of many compounds that might be involved in the above-described bioactivities.

Results of GC–MS analysis of all extracts analyzed by GC–MS revealed siloxane derivatives. In general, the compounds in the siloxane group are antibacterial [58,59]. For example, heptasiloxane, hexadecamethyl (detected in all analyzed extracts in this study) present in Egyptian red seaweed has antibacterial effect [60,61]. Cyclohexasiloxane, dodecamethyl- also detected in the profiles of extracts analyzed, has been reported as antimicrobial, antioxidant, antifungal, and emollient [61,62].

GC–MS analysis of ethyl acetate extract of *A. cavernicola* revealed butylphosphonic acid, 2-ethylhexyl propyl ester as a major compound with 37.468%. There is no activity reported about this compound specially, in the literature. But phosphonates were reported to be very useful in the therapeutic treatment due to the importance of the phosphonic substituents C-P(O)(OH)_2_, allowing for very remarkable flexibility for complexation with enzymes, on the one hand, and giving up the electrons or the hydrogen radicals through the process of electronic transfer attributed to this entity, on the other hand [63]. The second prevailing compound with 10.892% is 4-tert octylphenol, TMS derivative has no records of individual bioactivity. It was extracted from *Euphorbia hirta* leaf and is considered a novel bioactive compound [64]. The other compounds have a percentage of less than 10%. Moreover, a study by Sharma et al. (2009) [65] demonstrated the antimicrobial activities of imidazole derivatives against Gram-positive and Gram-negative bacteria, as well as fungi like *Candida albicans*. Other biological activities of imidazole and its derivatives included anti-inflammatory [66], anticancer [67], and antitubercular [68]. Hexadecanoic acid is one category of straight-chain saturated fatty acid commonly found in plants and animals. According to Pu et al. [69], hexadecanoic acid and its derivatives from neem oil could inhibit *S. aureus*, *E. coli*, and *Salmonella* sp. growth with MIC ranging from 20 to 0.625 mg/mL.

Many fungi have been reported to be producers of penicillic acid (2,5-Hexadienoic acid, 3-methoxy-5-methyl-4-oxo-), including the genus *Penicillium* and *Aspergillus* [70]. Since the penicillic acid was first isolated from the *Penicillium puberulum* [71], its various biological activities, such as antifungal, antitumor, antidiuretic, and various toxic activities, have been characterized [72,73]. In terms of antibacterial activity, penicillic acid is known to exert a strong inhibitory ability against Gram-positive and Gram-negative bacteria such as *Bacillus cereus*, *Streptococcus pneumoniae*, *P. aeruginosa*, and *S. aureus* [72]. 

Our study indicates, for the first time, the production of penicillic acid from *A. cavernicola*. *A. persii* is described as a member of section *Circumdati* with a strong production of penicillic acid [74]. In the work of Nguyen et al. (2016) [75], penicillic acid was isolated from *Aspergillus persii* EML-HPB1-11, and this compound effectively inhibited the growth of 12 plant pathogenic bacteria and successfully controlled bacterial spot disease on peach leaf. Obana et al.,1995, [76] reported the production of the polyketide 5,6-dihydropenicillic acid (DHPA) by various type strains of *Aspergillus ochraceus* and related strains of *Aspergillus* in nutrient cultures. This same study suggests that penicillic acid is produced on potato dextrose agar medium, but 5,6-dihydropenicillic acid is obtained under highly nutritious circumstances. It should be noted that both penicillic acid and 5,6-dihydropenicillic acid were obtained in our study following the cultivation of the *A. cavernicola* and *A. persii* in a potato dextrose broth. Imidazole derivatives possess antioxidant properties, expressed either by their capacity to scavenge free radicals or their ability to reduce lipid peroxidation [77].

Results of GC–MS analysis of ethyl acetate extract of *A. persii* revealed the furan 2,5-furandicarboxylic acid, tetrahydro-, dimethyl ester as a prevailing compound with (21.4686%). There is no activity reported about this compound in the literature. The second prevailing compound with (15.0705%) is the phosphinic acid, (1,1-dimethylethyl)[4-(1,1-dimethylethyl)phenyl]. Phosphinic acid compounds (phosphinates) are derivatives of phosphinic acid H_2_P(O)(OH) [78]. Phosphinic acid derivatives exhibit diverse biological activities (anti-inflammatory, anti-Alzheimer, antiparasitic, antihepatitis, antiproliferative, anti-influenza, anti-HIV, antimalarial, and antimicrobial) and a high degree of structural diversity, rendering them a versatile tool in the development of new medicinal agents [79]. The 2,5-hexadienoic acid, 3-methoxy-5-methyl-4-oxo- (penicillic acid) is the third prevailing (11.5193%); followed by l-Alanine, N-(3-fluorobenzoyl)-, heptyl ester (10.3966%). Other compounds detected in the ethyl acetate extract of *Aspergillus persii* were reported to have biological activity. For example, cyclopentane, 1-acetyl-1,2-epoxy- was reported to have anti-inflammatory, antiviral, and bronchodilatory properties [80,81]. Moon et al. (2010) [82] reported that Erythritol was a sweet antioxidant that released oxidative stress with unique nutritional properties. The study by Israa et al. (2017) [83] showed its anti-cariogenic effects. 3,4-Dihydroxymandelic acid, 4TMS derivative, was reported by Altameme et al. (2017) [84] as an antitumor, analgesic, and antimicrobial.

GC–MS analysis conducted on the crude ethyl acetate extract of *A. alternata* revealed tenuazonic acid (21.6718%); tetracosamethyl–cyclododecasiloxane (11.5408%); and bis (2˗ethylhexyl) phthalate (10.6541%) as major compounds. Tenuazonic acid has been used for the development of potential new drugs for the treatment of Alzheimer’s disease due to its acetylcholine esterase inhibitor and antioxidant properties, as well as its metal chelation capacity [85]. Tenuazonic acid produced by *A. alternata* showed a potential activity against *Mycobacterium tuberculosis* H37Rv [86]. Previous studies revealed that Tenuazonic acid is an efficient antibacterial, antiviral, and antitumor [87,88]. It is also qualified for its potential antifungal compounds, as described by Mousa et al., 2015, [89]. Tetracosamethyl-cyclododecasiloxane is reported as hepatoprotective, antispasmodic, and antirheumatic [90]. Our study reveals the production of the bis (2-ethylhexyl) phthalate by the genus *Alternaria*. It was found only in the subgenus *Penicillium* [91]. Bis-(2-ethylhexyl) phthalate (BEHP) has been reported as a potent bioactive secondary metabolite, naturally produced by bacterial, fungal, and algal species [92,93,94]. It has already been purified from the culture broth of a strain of actinomycetes *Nocardia levis* [95] and *Streptomyces bangladeshensis* [96]. These two studies indicate that the bis (2-ethylhexyl) phthalate exhibits antibacterial activity against bacterial strains *S. aureus*, *E. coli*, and *P. Aeruginosa*, among others. Recently, Bis-(2-ethylhexyl) phthalate (BEHP) was isolated from LAB species *Lactiplanti-bacillus plantarum* BCH-1, which showed significant larvicidal potential and antibacterial activity against *E. coli* and *S. aureus* [97]. The compound bis(2-ethylhexyl) phthalate, known as DEHP Di-(2-ethylhexyl) phthalate, was reported by Habib and Karim (2009) [98] to have antibacterial activity. Bis(2-ethylhexyl) phthalate is reported as an essential compound because of its biological properties as antioxidant, antiviral, and antitumor activity [93]. Our study reveals the production of 1,4-Benzenedicarboxylic acid, bis(2-ethylhexyl) ester (other used names = di(2-ethylhexyl) terephthalate, dioctyl terephthalate, bis-2-ethylhexyl terephthalate). It should be noted that DEHT and DEHP are phthalic and terephthalic acid esters, the main plasticizers that are used to confer elasticity and flexibility to various fiber and plastic products [99]. Many studies have identified the presence of phthalate in medicinal plants, which most often exhibited antimicrobial activities [100]. Tetratetracontane is an alkane with antibacterial properties [101]. Tert-hexadecanthiol plays an important role in antibacterial and antioxidant activities [102,103]. Furthermore, in the study by Acar et al. 2020 [104], a series of N-(2-hydroxy-(4 or 5)-nitrophenyl) benzamide derivatives were designed and synthesized. According to this study, the newly synthesized benzamide derivatives mainly exhibited significant antibacterial activities. Quinoline motifs are known to be free radical scavengers [105].

GC–MS method showed that 1,1,1,5,7,7,7-Heptamethyl-3,3-bis(trimethylsiloxy) tetra-siloxane (17.5764%); Cyclooctasiloxane, hexadecamethyl- (16.4283%); 3,4-Dihydroxymandelic acid, 4TMS derivative (12.4779%); and Heptasiloxane, hexadecamethyl-(11.1441%) were the most dominant volatile components in the *Z. lotus* extract. An anti-quorum sensing potential is reported about 1,1,1,5,7,7,7-Heptamethyl-3,3-bis(trimethylsiloxy)tetrasiloxane [106]. Cyclooctasiloxane, hexadecamethyl- is reported as antimicrobial [90], and Heptasiloxane, hexadecamethyl- has antibacterial effect [61]. Cytochalasins H showed potential antibacterial and cytotoxic activities [107]. On the other hand, hexanedioic acid, bis (2-ethylhexyl) ester, has been reported as an antifungal [108,109].

Moreover, the antioxidant activities and antibacterial activity of extracts may be related to the chemical compounds previously identified. They could also be the result of other unidentified compounds contained in the non-volatile phase or a synergistic effect of several compounds.

Interestingly, certain compounds are found in both types of extract (the endophytic fungal extracts and the host plant leaves extract). Endophytes have adapted themselves to their special microenvironments by genetic variation, including the uptake of some plant DNA into their own genomes [110]. After long-term coexistence with their host, endophytes can synthesize biologically active substances similar to the secondary metabolites produced by host plants [111,112,113]. Recent findings revealed that endophytic fungi, not their host plants, synthesize certain molecules [114,115], highlighting these microorganisms’ crucial role in the discovery of novel pharmacologically active compounds.

Few metabolites reported by our study have already been described in the literature concerning plant and fungal species. That can be explained by the difference in the fermentation conditions, the solvent used for extraction, and the detection method used. In fact, one approach to the efficient production of secondary metabolites is represented by fermentation, a microorganism-driven process that enzymatically converts substrates into bioactive compounds [116]. The fungal secondary metabolite production is affected by various parameters. Fermentation [117] and culture conditions could be modulated to optimize the production of specific bioactive molecules [118]. Extrolites, these small molecules produced by fungi, are particularly sensitive to environmental and nutritional conditions [5,119,120]. Their biosynthesis may sometimes require activation by biological or chemical stimulants [120,121,122,123]. The extraction method also affects the diversity of recovered secondary metabolites, with ethyl acetate showing particular efficiency as an extraction solvent [32]. Finally, the detection method of secondary metabolites is also important. Volatile metabolites can be separated and detected by GC–MS, whereas most other secondary metabolites are extracted by organic solvents and separated and detected by HPLC-DAD-MS (high-performance liquid chromatography with diode array detection coupled with a Mass Spectrometer) [124]. Among the volatile compounds synthesized by endophytes, some are already known and identified in essential oils or other phytochemical products, while others represent new molecules for which no standards are yet available [125,126]. Fungal identification has recently evolved through new approaches integrating the analysis of secondary metabolite profiles, complementing morphological and genetic characteristics [127]. These new data significantly enrich our knowledge of the studied fungi, particularly regarding *A. cavernicola*. This species, belonging to the series and section *Cavernicolarum*, is considered synonymous with *Aspergillus amylovorus* [128]. The section *Cavernicolarum* remains poorly explored [129], as evidenced by the incomplete characterization of metabolites produced by this strain, of which only asperugin (a phenolic polyketide), red pigments related to monascorubramins, and some metabolites of the undetermined structure are currently identified [127].

## 5. Conclusions

In this study, the antibacterial and antioxidant activities of ethyl acetate extracts of leaves of *Z. lotus* and five endophytic fungi associated (*C. asperulatum*, *A. cavernicola*, *A. persii*, *A. alternata*, and *F. incarnatum–equiseti complex)* were revealed.

*A. cavernicola* demonstrated the most promising antibacterial activity, exhibiting broad-spectrum inhibition against both Gram-positive and Gram-negative bacteria, with particularly strong efficacy against *Staphylococcus aureus* (including the hospital-acquired MRSA strain), *Enterococcus faecalis*, and *Escherichia coli*. The antibacterial activity of *A. cavernicola* was not only significant in terms of inhibition zone sizes but also displayed low MIC values, indicating its strong antimicrobial potential.

The antioxidant potential was evaluated through DPPH and reducing power assays. *A. cavernicola* again showed the most potent antioxidant activity, suggesting it could serve as a valuable source of natural antioxidants. In contrast, other fungal species like *C. asperulatum* and *F. incarnatum–equiseti complex* showed limited or no antioxidant activity, indicating that not all endophytic fungi from *Z. lotus* possess significant antioxidant properties.

Additionally, the phenolic content analysis revealed that *A. cavernicola* contained the highest concentration of total phenolics, which likely contributes to both its antibacterial and antioxidant activities. The phenolic content in the *A. cavernicola* extract was nearly double that of *Z. lotus* leaves, suggesting a synergistic relationship between the host plant and its endophytic fungi in the biosynthesis of bioactive compounds.

The GC–MS analysis of the fungal extracts further confirmed the presence of a wide variety of bioactive compounds. Our study indicates, for the first time, the production of penicillic acid (2,5-Hexadienoic acid, 3-methoxy-5-methyl-4-oxo-) from *A. cavernicola.* Similarly, *A. persii* and *A. alternata* extracts contained unique compounds, some of which have been previously associated with antimicrobial and antioxidant properties, further supporting the potential of these fungi in drug discovery.

In summary, this study underscores the significant antimicrobial and antioxidant potential of endophytic fungi from *Z. lotus*, particularly *A. cavernicola*, which exhibit both broad-spectrum antibacterial activities and potent antioxidant properties. The diversity of bioactive compounds identified through GC–MS highlights the complex biochemical profiles of these fungal extracts, suggesting their promise as natural sources of therapeutic agents. Further research is needed to isolate and characterize these compounds in greater detail, as well as to evaluate their in vivo efficacy and safety, to fully exploit the potential of *Z. lotus* endophytic fungi for pharmaceutical and biotechnological applications.

## Figures and Tables

**Table 1:** Bacterial growth inhibition: Measuring effectiveness of leaf and fungal extracts.

Extracts	Inhibition Zones (mm ± ET) Corresponding to Each Bacteria				
	*Staphylococcus aureus* ATCC 25923	*Staphylococcus aureus* MU50	*Enterococcus faecalis* WDCM00009	*Escherichia coli* ATCC 25922	*Pseudomonas aeruginosa* ATCC 27853 ATCC 27853
*Ziziphus lotus*	8 ± 00	8 ± 00	00 ± 00	00 ± 00	00 ± 00
*Fusarium incarnatum–equiseti complex*	20 ± 00	20 ± 00	20 ± 00	15 ± 1	00 ± 00
*Aspergillus cavernicola*	32 ± 00	31.33 ± 1.15	30 ± 00	25 ± 00	9 ± 00
*Aspergillus persii*	27 ± 1	27 ± 1	26 ± 00	17.33 ± 2	00 ± 00
*Cladosporium asperlatum*	23.33 ± 1.15	21.66 ± 1.52	24 ± 00	21 ± 2	00 ± 00
*Alternaria alternata*	27 ± 1	27 ± 1	25 ± 1	24.66 ± 12	00 ± 00
DMSO	00 ± 00	00 ± 00	00 ± 00	00 ± 00	00 ± 00
Chloramphenicol	35.66 ± 4.61	31 ± 1	25 ± 00	25 ± 00	12 ± 1

**Table 2:** MIC and MBC of the five endophytic fungi crude extracts against tested pathogenic bacteria.

Extracts	*Staphylococcus aureus* ATCC 25923		*Staphylococcus aureus* MU50		*Enterococcus faecalis* WDCM00009		*Escherichia coli ATCC 25922*		*Pseudomonas aeruginosa* ATCC 27853	
	MIC	MBC	MIC	MBC	MIC	MBC	MIC	MBC	MIC	MBC
*Fusarium incarnatum–equiseti complex*	1.25	>5	1.25	>5	1.25	>5	0.625	>5	-	-
*Aspergillus cavernicola*	0.078	1.25	0.312	1.25	0.078	1.25	0.078	5	0.625	2.5
*Aspergillus persii*	0.312	5	1.25	>5	0.078	2.5	0.312	2.5	-	-
*Cladosporium asperlatum*	1.25	5	2.5	>5	5	>5	0.312	5	-	-
*Alternaria alternata*	0.312	5	0.312	2.5	0.625	2.5	0.625	>5	-	-

**Table 3:** Compounds identified by GC–MS analysis.

N°	Compound Name	Area Percent			
		*Z. lotus*	*A. Cavernicola*	*A. Persii*	*A.* *Alternata*
1	Compounds found in the four extracts analyzed	5.7404	0.9913	0.907	4.7928
	Cyclohexasiloxane, dodecamethyl-				
2	Octasiloxane, 1,1,3,3,5,5,7,7,9,9,11,11,13,13,15,15-hexadeca methyl-	4.7606	2.5001	0.6781	6.7681
3	Tetracosamethyl-cyclododecasiloxane	8.2217	2.4954	2.8193	11.5408
4	Heptasiloxane, hexadecamethyl-	11.1441	2.1758	4.4951	0.876
5	1,1,1,5,7,7,7-Heptamethyl-3,3-bis(trimethylsiloxy)tetrasiloxane	17.5764	2.8554	5.0515	7.376
6	Cyclononasiloxane, octadecamethyl-	2.0831	0.7289	2.0313	8.2317
7	Compounds found in only three extracts	12.4779	1.0161	1.1417	-
	3,4-Dihydroxymandelic acid, 4TMS derivative				
8	Cyclooctasiloxane, hexadecamethyl-	16.4283	-	4.1513	9.5733
9	Compounds found in only two extracts	1.3423	-	0.6849	-
	Hexanedioic acid, bis(2-ethylhexyl) ester				
10	3,4-Dihydroxyphenylglycol, 4TMS derivative	3.5736	-	-	0.1525
11	Heptasiloxane, 1,1,3,3,5,5,7,7,9,9,11,11,13,13- tetradecamethyl-	6.3661	-	-	2.2806
12	Penicillic acid (2,5-Hexadienoic acid, 3-methoxy-5-methyl-4-oxo-	-	0.5054	11.5193	-
13	Succinic acid, ethyl pent-4-en-2-yl ester	-	0.8701	0.5604	-
14	5,6-Dihydropenicillic acid	-	7.1999	0.6153	-
15	Cholestan-5-en-3-ol piperidinomethyl ether	-	6.13	1.4362	
16	Phosphinic acid, (1,1-dimethylethyl)[4-(1,1-dimethylethyl)phenyl]-	-	4.6934	15.0705	-
17	Compounds found in only one extract	0.3478	-	-	-
	Cytochalasin H				
18	6-chlorohexanoic acid 3-methylbuttyl ester	0.459	-	-	-
19	Furan, 2-(dichloromethyl)-tetrahydro-	0.948	-	-	-
20	Hexanoic acid, 2-methyl-	0.2581	-	-	-
21	Phthalic acid, 6-ethyl-3-octyl butyl ester	1.1848	-	-	-
22	2,5-Dihydroxybenzoic acid, 3TMS derivative	6.1423	-	-	-
23	2,3-Dimethyl-2-heptene	-	8.4199	-	-
24	Hexadecanoic acid, 1-[[[(2-aminoethoxy) hydroxy phosphinyl]oxy]methyl]-1,2-ethanediyl ester	-	0.6677	-	-
25	5-Hydroxy-4-methoxy-5-(prop-1-en-2-yl)furan-2(5H)-one	-	0.5835	-	-
26	2-Nonen-4-one	-	1.4811	-	-
27	2-(4-Ethoxyanilino)-N-propylpropanamide, Ac derivative	-	1.0839	-	-
28	Butylphosphonic acid, 2-ethylhexyl propyl ester	-	37.468	-	-
29	4-tert-Octylphenol, TMS derivative	-	10.892	-	-
30	1H-Imidazole, 1-(1-oxooctadecyl)-	-	0.5937	-	-
31	2-(1-Methylpiperidin-2-yl) ethanol	-	0.7136	-	-
32	4-(4-Hydroxyphenyl)-4-methyl-2-pentanone, TMS derivative	-	0.3224	-	-
33	Sydowinin A, 2TMS derivative	-	1.322	-	-
34	Hexanedioic acid, dioctyl ester	-	0.4746	-	-
35	Phenanthrene-10-ethanamine, 3-bromo-á-hydroxy-N,N-diheptyl-, O,O-diphenylphosphate	-	0.3118	-	-
36	Cyclodecasiloxane, eicosamethyl-	-	1.624	-	-
37	Cyclopentane, 1-acetyl-1,2-epoxy-	-	-	0.4226	-
38	Flucytosine	-	-	1.6921	-
39	2-Benzyl-3-methoxycyclopropanecarboxylic acid, methyl ester	-	-	1.0194	-
40	Succinic acid, ethyl 4-methylhept-3-yl ester	-	-	1.8956	-
41	5-Hydroxy-4-methoxy-3-(1-methoxypropan-2-yl)furan-2(5H)-one	-	-	0.3608	-
42	Erythritol	-	-	5.3667	-
43	2,5-Furandicarboxylic acid, tetrahydro-, dimethyl ester	-	-	21.4686	-
44	Epinephrine, (á)-, 3TMS derivative	-	-	0.7011	-
45	l-Alanine, N-(3-fluorobenzoyl)-, heptyl ester	-	-	10.3966	-
46	Phthalic acid, butyl hex-3-yl ester	-	-	0.4355	-
47	Ethene-1,1-diamine, 2,2-dinitro-	-	-	0.5094	-
48	3-Cholestanol piperidinomethyl ether	-	-	0.4484	-
49	Pentanedioic acid, 1-(6-bromo-9-phenanthrenyl)-2-(diheptylamino)ethyl monoester	-	-	0.3404	-
50	Hexasiloxane, tetradecamethyl-	-	-	2.5974	-
51	Tenuazonic acid	-	-	-	21.6718
52	Tetratetracontane	-	-	-	0.1631
53	8-Cinnamoyl-5,7-dihydroxy-2,2,6-trimethylchromene, 2TMS derivative	-	-	-	2.8126
54	Octyl tetracosyl ether	-	-	-	1.4379
55	tert-Hexadecanethiol	-	-	-	0.921
56	Hexadecane, 1-bromo-	-	-	-	0.5355
57	Heptacosane	-	-	-	0.8685
58	Terephthalic acid, bis(2,2,3,3,4,4,5,5,6,6,7,7-dodecafluoroheptyl) ester	-	-	-	1.7433
59	4-Hydroxybenzyl alcohol, 2TBDMS derivative	-	-	-	0.7761
60	2-(5-Bromo-pyridin-2-ylamino)-3,3,3-trifluoro-2-(4-methoxy-benzoylamino)-propionic acid methyl ester	-	-	-	1.1012
61	N-(2-Hydroxy-4-nitrophenyl)-4-methoxybenzamide, TMS derivative	-	-	-	0.3732
62	Octadecane, 1-iodo-	-	-	-	0.531
63	Hexadecane	-	-	-	0.2867
64	Bis(2-ethylhexyl) phthalate	-	-	-	10.6541
65	[1,3]-Oxazino[5,6-c]quinoline,3-(3,4-methylenedioxybenzyl)-5-trifluoromethyl-3,4(2H)-dihydro-7-methoxy-	-	-	-	0.2634
66	para-Isopropylbenzoic acid trimethylsilylester	-	-	-	0.5585
67	Dodecane, 1-iodo-	-	-	-	0.2764
68	1,4-Benzenedicarboxylic acid, bis(2-ethylhexyl) ester	-	-	-	0.3119
69	Dodecane, 3-methyl-	-	-	-	0.0976
70	Supraene	-	-	-	1.2095
71	Phenol, 2-amino-4,6-bis (1,1-dimethylethyl)-	-	-	-	0.3795
72	Benzene, 1,1,1-[1-(bromomethyl)-2-methoxy-1-methyl-1-ethanyl-2-ylidene]tris-	-	-	-	0.9267

## Data Availability

The original contributions presented in this study are included in this article; further inquiries can be directed to the corresponding author.
